# Resveratrol can enhance osteogenic differentiation and mitochondrial biogenesis from human periosteum-derived mesenchymal stem cells

**DOI:** 10.1186/s13018-020-01684-9

**Published:** 2020-06-03

**Authors:** Dong Kyu Moon, Bo Gyu Kim, A Ram Lee, Yeong In Choe, Imran Khan, Kyoung Mi Moon, Ryoung-Hoon Jeon, June-Ho Byun, Sun-Chul Hwang, Dong Kyun Woo

**Affiliations:** 1 Department of Orthopedic Surgery and Institute of Health Sciences, School of Medicine and Gyeongsang National University Hospital, Gyeongsang National University, Jinju, Republic of Korea; 2 College of Pharmacy and Research Institute of Pharmaceutical Sciences, Gyeongsang National University, Jinju, Republic of Korea; 3 Department of Theriogenology and Biotechnology, College of Veterinary Medicine, Gyeongsang National University, Jinju, Republic of Korea; 4 Department of Oral and Maxillofacial Surgery and Institute of Health Sciences, School of Medicine and Hospital, Gyeongsang National University, Jinju, Republic of Korea

**Keywords:** Mesenchymal stem cell, Resveratrol, Osteogenesis, Mitochondria

## Abstract

**Background:**

Osteoporosis is a metabolic bone disorder that leads to low bone mass and microstructural deterioration of bone tissue and increases bone fractures. Resveratrol, a natural polyphenol compound, has pleiotropic effects including anti-oxidative, anti-aging, and anti-cancer effects. Resveratrol also has roles in increasing osteogenesis and in upregulating mitochondrial biogenesis of bone marrow-derived mesenchymal stem cells (BM-MSCs). However, it is still unclear that resveratrol can enhance osteogenic differentiation or mitochondrial biogenesis of periosteum-derived MSCs (PO-MSCs), which play key roles in bone tissue maintenance and fracture healing. Thus, in order to test a possible preventive or therapeutic effect of resveratrol on osteoporosis, this study investigated the effects of resveratrol treatments on osteogenic differentiation and mitochondrial biogenesis of PO-MSCs.

**Methods:**

The optimal doses of resveratrol treatment on PO-MSCs were determined by cell proliferation and viability assays. Osteogenic differentiation of PO-MSCs under resveratrol treatment was assessed by alkaline phosphatase activities (ALP, an early biomarker of osteogenesis) as well as by extracellular calcium deposit levels (a late biomarker). Mitochondrial biogenesis during osteogenic differentiation of PO-MSCs was measured by quantifying both mitochondrial mass and mitochondrial DNA (mtDNA) contents.

**Results:**

Resveratrol treatments above 10 μM seem to have negative effects on cell proliferation and viability of PO-MSCs. Resveratrol treatment (at 5 μM) on PO-MSCs during osteogenic differentiation increased both ALP activities and calcium deposits compared to untreated control groups, demonstrating an enhancing effect of resveratrol on osteogenesis. In addition, resveratrol treatment (at 5 μM) during osteogenic differentiation of PO-MSCs increased both mitochondrial mass and mtDNA copy numbers, indicating that resveratrol can bolster mitochondrial biogenesis in the process of PO-MSC osteogenic differentiation.

**Conclusion:**

Taken together, the findings of this study describe the roles of resveratrol in promoting osteogenesis and mitochondrial biogenesis of human PO-MSCs suggesting a possible application of resveratrol as a supplement for osteoporosis and/or osteoporotic fractures.

## Background

Elderly people occupy the fastest-growing segment of populations throughout the world. Accordingly, age-associated bone diseases such as osteoporosis have been increasing. Due to low mineral density and concomitant change of microarchitecture of the bone tissue, osteoporosis manifests as an increased risk of bone fracture [1]. The lifetime risk for an osteoporotic hip, spine, or forearm fracture at the age of 50 years has been estimated to be 40~53% in women and 13~22% in men [2]. These osteoporotic fractures are not only problematic in the fracture itself, but they also exacerbate the patient’s medical condition, affect mortality, and increase socioeconomic costs [3, 4]. Therefore, many attempts aimed at diagnosis and treatment for osteoporosis have been conducted to prevent osteoporotic fracture.

Adult mesenchymal stem cells (MSCs) are multipotent stem cells capable of differentiating into various cell types including adipocyte, chondrocyte, and osteocyte [5]. In addition, adult MSCs have some benefits regarding immunologic and ethical issues relative to embryonic stem cells. Thus, MSCs have been actively applied as a material for regenerative medicine for osteoporosis treatment [6–8]. Traditionally, bone marrow-derived MSCs (BM-MSCs) have been widely chosen as a stem cell source in regenerative medicine [9]. However, BM-MSCs also have some limitations. For example, BM-MSC isolation needs bone marrow aspiration which is painful and has a risk of infection. Furthermore, BM-MSCs from older bone marrows were reported to have decreased expansion and differentiation potentials [10, 11]. Unfortunately, there is a report that BM-MSCs do not make much contributions to fracture healing [12]. Besides the bone marrow, the periosteum also contains adult MSCs. These periosteum-derived MSCs (PO-MSCs) are known to play an important role in fracture healing [12]. Moreover, PO-MSCs seem to maintain their differentiation potentials even with increasing age [13, 14]. These advantages make PO-MSCs an attractive MSC source in regenerative medicine for the bone [15].

Recently, the metabolic shift from glycolysis to mitochondrial oxidative phosphorylation (OXPHOS) is taking on increased importance in stem cell differentiation [16, 17]. Glycolytic metabolism is known as the main metabolism for self-renewal and maintenance in proliferating stem cells. However, when stem cells commit to differentiate, glycolytic metabolism is shifted to mitochondrial OXPHOS to meet an increased cellular energy demand in differentiated cells [18, 19]. OXPHOS occurs within the mitochondria where the mitochondrial respiratory complexes and the ATP synthase produce ATP. Catalytic core subunits of mitochondrial respiratory complexes as well as the ATP synthase are encoded in mitochondrial DNA (mtDNA). Thus, if cells require more ATP during stem cell differentiation, cells need to upregulate mitochondrial biogenesis leading to an increased cellular mitochondrial mass and mtDNA contents.

Resveratrol is a natural polyphenol found in red grapes, peanuts, berries, pomegranates, etc. [20, 21]. Resveratrol has pleiotropic effects including anti-oxidative, anti-aging, and anti-cancer effects [22–25]. Among these diverse effects of resveratrol, one notable benefit of resveratrol is to increase mitochondrial biogenesis in mammalian cells [26, 27]. Moreover, resveratrol also promotes osteogenic differentiation of bone marrow- or adipose tissue-derived mesenchymal stem cells [28, 29]. However, so far, it is still unclear that resveratrol can enhance osteogenic differentiation or mitochondrial biogenesis of PO-MSCs which play important roles in bone fracture healing. Thus, this study investigated whether resveratrol has a role in osteogenic differentiation and mitochondrial biogenesis of PO-MSCs.

## Materials and methods

### Reagents, in vitro cell cultures, and osteogenesis of human periosteum-derived mesenchymal stem cells

All chemicals used in this study were purchased from Sigma-Aldrich (St. Louis, MO, USA). Cell culture media and fetal bovine serum were purchased from Invitrogen (Waltham, MA, USA). Human periosteal tissues were obtained from patients who granted informed consent for the collection of the tissues, as required by the Ethics Committee of Gyeongsang National University Hospital (GNUH 2014-05-012). PO-MSCs were then isolated as described previously [19]. Briefly, periosteal explants were harvested from the mandibles during surgical extraction of the impacted lower third molars. Periosteal pieces were washed and cultured at 37 °C, 95% humidified air, and 5% CO_2_ in 100-mm culture dishes containing Dulbecco’s modified Eagle’s medium (DMEM) supplemented with 10% heat-inactivated fetal bovine serum, 100 IU/mL penicillin, and 100 μg/mL streptomycin. Resulting adherent cells were passaged by gentle trypsinization and reseeding in a fresh medium. The cell culture medium was changed every 3 days during the isolation period. For osteogenic differentiation, PO-MSCs were cultured using osteogenesis induction medium (OM) which is composed of DMEM, supplemented with 50 μg/mL l-ascorbic acid 2-phosphate, 10 nM dexamethasone, and 10 mM β-glycerophosphate [19].

### Assessment of PO-MSC proliferation and viability under resveratrol treatments

The proliferation of PO-MSCs was measured by cell counting. Briefly, 2 × 10^4^ cells were seeded in 24-well plates and were cultured in OM medium. Resveratrol treatment was performed by treating cells with vehicle (ethanol) or a range of resveratrol concentrations from 500 nM up to 20 μM. Five-day and 10-day cultures were trypsinized, and resulting detached cells were counted with a hemocytometer. The viability of PO-MSCs treated with resveratrol was determined by MTT assays. Shortly, 2 × 10^4^ cells were seeded in 24-well plates and cultured in OM medium with resveratrol treatments (500 nM~20 μM). Five-day and 10-day cultures were subjected to a colorimetric MTT assay.

### Measurements of alkaline phosphatase activities

Alkaline phosphatase (ALP) activities were determined colorimetrically using an ALP Assay Kit (Takara, Kusatsu, Japan) according to the manufacturer’s instructions. In brief, whole-cell lysates were prepared using a NP-40 lysis buffer (Thermo Scientific, Waltham, USA). Cell lysates were then incubated with p-nitrophenylphosphate, a colorless substrate for ALP, in a Tris-HCl buffer (pH 9.5) at 37 °C for 15 min. Upon splitting off the phosphate group of p-nitrophenylphosphate by ALP, p-nitrophenol is released and detected spectrophotometrically (absorption maximum, 405 nm). These ALP activities then were normalized to cellular protein contents determined by the Bradford method.

### Measurements of calcium deposits

Mineralization of PO-MSC cultures with resveratrol treatments (500 nM and 5 μM) in the OM medium was assessed by von Kossa staining [30]. Calcium contents during osteogenesis of PO-MSCs were also measured by a calcium deposition assay. In brief, at days 14 and 21 of cell cultures, PO-MSCs grown in OM medium with resveratrol treatments (500 nM and 5 μM) were decalcified with 0.6-N HCl for 1 day at room temperature. Then, the calcium content was determined by the colorimetrical o-cresolphthalein complexone method (Calcium C-test, Wako Chemicals, Japan), whereby calcium reacts with o-cresolphthalein to form a purple complex that absorbs light with a wavelength of 570 nm. After decalcification, the total protein content in the supernatants was measured by the Bradford method and was used to normalize calcium content.

### Measurements of mitochondrial mass by flow cytometry and fluorescent microscopy

Mitochondrial mass of cultured PO-MSCs was quantified by flow cytometry. Briefly, 1 × 10^5^ cells were stained in PBS with MitoTracker Green FM dye (Invitrogen, Waltham, MA, USA) for 30 min, washed, and resuspended in 200 μL PBS with 1% fetal bovine serum. Cellular fluorescence signals were then analyzed using a FACSCalibur (BD Biosciences, San Jose, CA, USA). Resulting flow cytometry data were analyzed using the FlowJo software version 8.7.3 (Ashland, OR, USA). For fluorescent microscopy, PO-MSCs cultured on chamber slides were stained with MitoTracker Green FM dye. Cellular mitochondria were visualized under a fluorescent microscope (Zeiss Axio Observer Z1, Carl Zeiss, Oberkochen, Germany), and images were analyzed using the ImageJ software (NIH, Bethesda, CA, USA).

### mtDNA copy number analysis by quantitative PCR

For mtDNA copy number analysis, total cellular DNA was extracted from cultured PO-MSCs. A quantitative real-time PCR method was used to determine the relative abundance of mtDNA versus nuclear 18S rDNA using corresponding mitochondrial and nuclear PCR primer sets in two parallel PCR reactions as described previously [19]. Relative mtDNA copy number was calculated as the ratio of the amount of amplification obtained with mtDNA versus nuclear 18S rDNA PCR primer sets for each sample and plotted normalized to the control group. The sequence of the PCR primer pairs is as follows: the nuclear 18S rRNA fragment was amplified by the primer pair 5′-TAGAGGGACAAGTGGCGTTC-3′ and 5′-CGCTGAGCCAGTCAGTGT-3′, and the mitochondrial COX1 fragment was amplified by the primer pair 5′-CACCCAAGAACAGGG TTTGT-3-3′ and 5′-TGGCCATGGGTATGTTGTTAA-3′.

### Statistical analysis

All experiments were performed using at least three independent cell cultures. Error bars in all figures represent the mean ± SEM, and statistical analyses were computed using the GraphPad Prism 7 software (GraphPad, San Diego, CA, USA). Student’s two-tailed *t* test was used for the determination of the statistical significance between the groups, and a *p* value of < 0.05 was considered statistically significant.

## Results

### Resveratrol treatments affect neither PO-MSC proliferation nor its viability

**Fig. 1:**
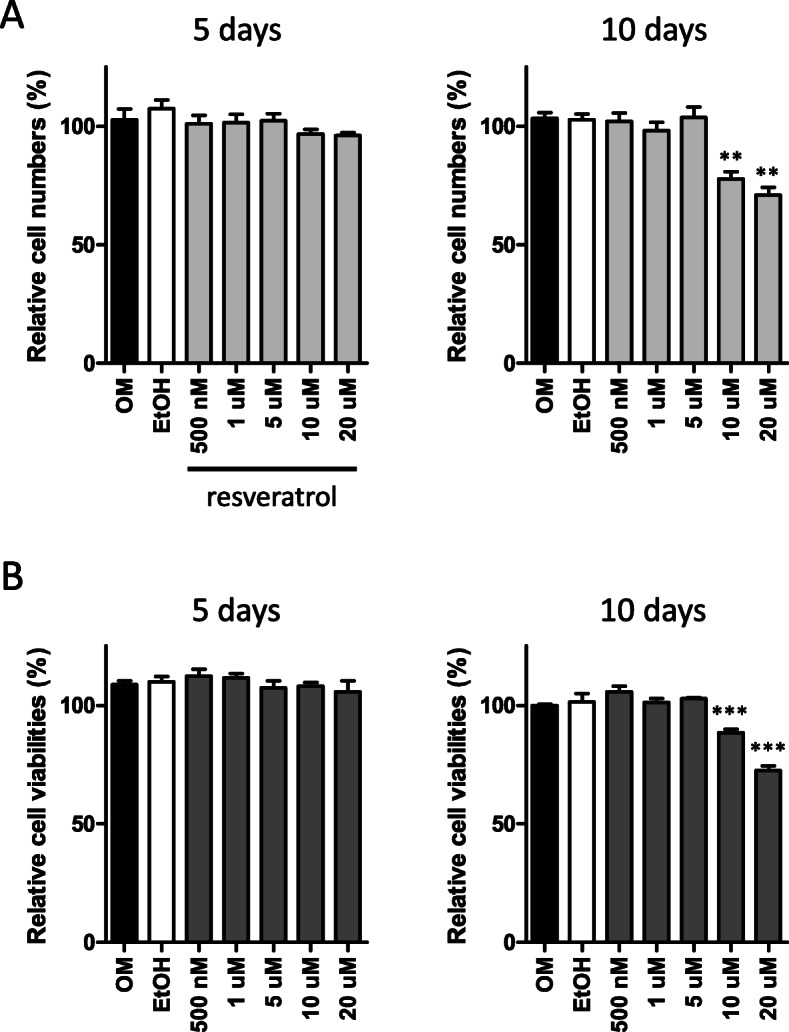
Effects of resveratrol on PO-MSC proliferation and viability. **a** Cell proliferation of PO-MSCs treated with resveratrol under the OM medium for 5 and 10 days was measured by cell counting. **b** Cell viability of PO-MSCs treated with resveratrol under OM medium for 5 and 10 days was measured by MTT assay

In order to see the effects of resveratrol on cell proliferation and viability during osteogenesis, PO-MSCs were cultured in the OM medium for 5 and 10 days. For the same periods of cultures, these PO-MSCs were also treated with various concentrations of resveratrol from 500 nM up to 20 μM. At day 5 and day 10 cultures, cells were harvested and counted with a hemocytometer for cell proliferation or were subjected to an MTT assay for cell viability. As shown in Fig. 1a, 500 nM, 1 μM, and 5 μM resveratrol treatments do not affect PO-MSC proliferation for 5 and 10 days of osteogenic cultures compared to untreated or vehicle (ethanol) controls. However, 10 μM and 20 μM resveratrol treatments for 10 days decreased PO-MSC proliferation by about 30% relative to controls. Similarly, in Fig. 1b, regarding cell viability during osteogenic cultures of PO-MSCs, lower concentrations of resveratrol treatments (500 nM, 1 μM, and 5 μM) had no effects, but higher concentrations of resveratrol (10 μM and 20 μM) decreased PO-MSC viability by up to 20% compared to controls. These results indicate that resveratrol treatments below 5 μM do not alter PO-MSC proliferation and viability during osteogenic cell cultures for at least 10 days. From these results, two different concentrations of resveratrol (500 nM and 5 μM) were chosen for the subsequent experiments in this study.

### Resveratrol treatments increase ALP activities in PO-MSCs during osteogenesis

**Fig. 2:**
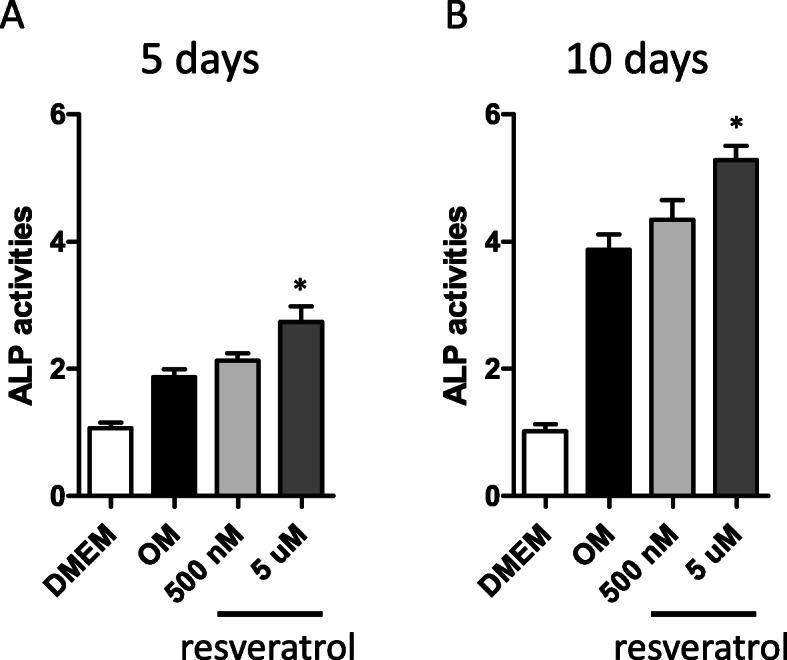
Effects of resveratrol on ALP activity in PO-MSCs during osteogenesis. ALP activity with resveratrol treatments (500 nM and 5 μM) for 5 and 10 days in OM medium was measured

An increase of ALP activity is known as an early biomarker for osteogenic differentiation or osteoblast activity. To test an effect of resveratrol on osteogenic differentiation of PO-MSCs, ALP activities were assessed by a colorimetric assay described in the “Methods” section. Figure 2 shows the ALP activities of PO-MSCs triggered to differentiate into osteoblast lineage for 5 and 10 days. Compared to undifferentiated PO-MSCs grown in DMEM, ALP activities were clearly increased in PO-MSCs undergoing osteogenic differentiation by OM culture conditions indicating that OM induces osteogenesis in PO-MSCs. The increase of ALP activity in osteogenically differentiating PO-MSCs is further enhanced by resveratrol treatments (500 nM and 5 μM) in time- and dose-dependent manners relative to untreated controls although 500 nM resveratrol treatment did not show a statistical significance. These results indicate that resveratrol can promote osteogenic differentiation of PO-MSCs.

### Resveratrol treatments promote mineralization in osteogenic cultures of PO-MSCs

**Fig. 3:**
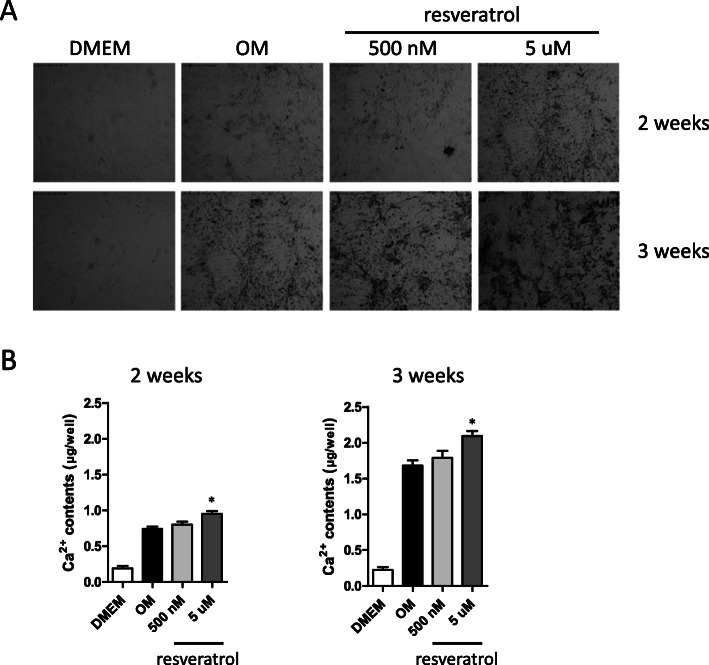
Effects of resveratrol on calcium deposit levels of PO-MSCs during osteogenesis. **a** von Kossa staining of PO-MSCs at 2 and 3 weeks of osteogenic cultures. **b** Calcium contents (μg/well) in osteogenic cultures (2 and 3 weeks) of PO-MSCs determined by a chromogenic assay

Mineralization by calcium deposits is known as a late biomarker for osteogenesis. In order to test if resveratrol can bolster mineralization during osteogenic differentiation, PO-MSCs were cultured in OM conditions for 2 and 3 weeks with or without resveratrol in the culture medium. From von Kossa staining images which mark mineralization shown in Fig. 3a, OM condition led to more mineralization in PO-MSC cultures compared to the control DMEM condition. This mineralization is further increased by resveratrol treatments (500 nM and 5 μM) in time- and dose-dependent manners relative to untreated OM conditions. In addition, colorimetric quantitation assays for calcium contents in cell cultures showed that calcium contents in PO-MSCs were increased by OM condition relative to DMEM condition, and this increase was further enhanced by resveratrol treatments (Fig. 3b). Together, these results demonstrate that 500 nM and 5 μM resveratrol treatments can enhance matrix mineralization during osteogenic differentiation of PO-MSCs.

### Resveratrol treatments upregulate mitochondrial biogenesis during the osteogenic differentiation of PO-MSCs

**Fig. 4:**
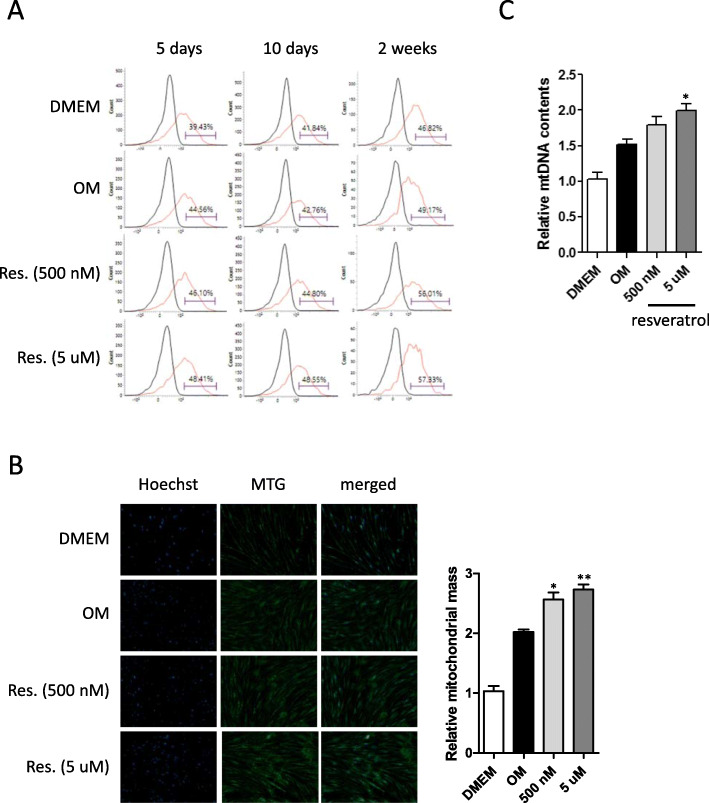
Effects of resveratrol on mitochondrial biogenesis in PO-MSCs during osteogenesis. **a** Analysis of mitochondrial mass by flow cytometry (black line, unstained; red line, Mitotracker Green FM (MTG) stained). **b** Fluorescence microscopy of PO-MSCs at 2 weeks of osteogenic cultures using MTG staining and quantification. **c** mtDNA contents determined by qPCR

Recently, the upregulation of mitochondrial biogenesis has been reported as a characteristic of differentiated MSCs. Moreover, it is well known that resveratrol has a beneficial effect on mitochondrial biogenesis in various cell types. Thus, to test whether mitochondrial biogenesis is upregulated by resveratrol during osteogenic differentiation of PO-MSCs, mitochondrial mass and mtDNA contents were measured. PO-MSCs were cultured and induced to osteogenic differentiation by OM with or without resveratrol treatments. The resulting differentiated PO-MSCs were stained with a fluorescent dye, Mitotracker Green FM (Invitrogen), which is localized in the mitochondria in the cell. The stained cells were then assessed for mitochondrial mass by flow cytometry. In Fig. 4a, compared to undifferentiated DMEM controls, OM increased mitochondrial mass and this increase was further enhanced by resveratrol treatments in time- and dose-dependent manners. In addition, from fluorescent microscopic images at 2 weeks of osteogenic cultures of PO-MSCs, resveratrol increased mitochondrial mass in a dose-dependent manner (Fig. 4b). Furthermore, mtDNA contents measured by qPCR were also increased by resveratrol treatments in osteogenically differentiated PO-MSCs relative to controls (Fig. 4c). Together, these findings clearly showed that mitochondrial biogenesis can be upregulated by resveratrol during the osteogenic differentiation of PO-MSCs.

## Discussion

In this study, we demonstrated that resveratrol treatments (5 μM) upregulate both ALP activities and calcium deposits during osteogenic differentiation of PO-MSCs compared to the untreated controls. These results indicate that resveratrol can enhance osteogenic differentiation of PO-MSCs. In other studies, resveratrol was reported to promote osteogenic differentiation of bone marrow- or adipose tissue-derived MSCs at higher concentrations [28–32]. However, in this study, PO-MSC viability decreased when the resveratrol dose was above 10 μM. It seems that the osteogenic effect of resveratrol varies according to the dosage or cell types [33–37]. Therefore, further investigation on the optimal dosage and administration method of resveratrol for accelerating osteogenesis of PO-MSC is needed.

In addition, this study showed that resveratrol treatments increase both mitochondrial mass and mtDNA contents during osteogenic differentiation in PO-MSCs. These findings indicate that resveratrol can upregulate mitochondrial biogenesis during osteogenic differentiation of PO-MSCs and are consistent with other reports regarding mitochondrial biogenesis by resveratrol in various cell types [26, 38–42]. Moreover, the upregulation of mitochondrial biogenesis by resveratrol in PO-MSCs is in line with the notion that metabolic shift from glycolysis to mitochondrial OXPHOS occurs during stem cell differentiation [16, 17, 43–47]. Thus, these results suggest that a small molecule upregulating mitochondrial biogenesis such as resveratrol can be a new modulator that may enhance osteogenesis in adult PO-MSCs for regenerative medicine.

Currently, the most widely used therapeutic agents for osteoporosis are anti-resorptive drugs such as bisphosphonate and selective estrogen receptor modulators (SERMs) [48]. However, these medications can cause complications when used for a long time. In particular, bisphosphonate has complications such as gastrointestinal irritation, atypical fracture due to decreased bone replacement rate, and osteonecrosis of the jaw bone [49–52]. In addition, venous thromboembolism can occur as a complication when using SERMs [53, 54]. On the other hand, a bone anabolic agent, teriparatide (a parathyroid hormone analog), has recently been used for osteoporosis treatment. But its disadvantages are the high cost, the need for injection, and the possibility of developing malignant tumors over a long period of time [55]. Thus, there is an increasing demand for a new bone anabolic agent. Interestingly, recent studies have shown that oral intake of adequate amounts of resveratrol can prevent bone fractures [56, 57]. In this regard, this study suggests that resveratrol supplements may be clinically meaningful in increasing bone tissue formation in the osteoporotic bones.

Fracture healing in the osteoporotic bone is poor compared to fractures occurring at a young age. For example, the fixation failure or nonunion rates of osteoporotic fractures were reported to be approximately 15~24.9% depending on specific fractures [58, 59]. Cellular factors contributing to delayed healing can be a decreased number of MSCs and/or osteoblasts with increasing age as well as an impaired response of bone cells in osteoporotic bone [60]. Therefore, regenerative medicine utilizing adult stem cells has been spotlighted in the prevention and treatment of osteoporosis and osteoporotic fracture [6–8, 61, 62]. However, BM-MSCs have some disadvantages, for example, a decreased cellular expansion and differentiation potentials from older bone marrow tissues. These limitations hinder BM-MSCs from their therapeutic application [10–12]. Alternatively, PO-MSCs derived from the periosteum of the bone have some benefits. Because they locate in the periosteum, it is expected that they are easy to participate in bone tissue maintenance in vivo. Moreover, the expansion and differentiation potentials of PO-MSCs are maintained even in older ages [13, 14]. Thus, PO-MSCs were expected to play a more critical role in bone healing than BM-MSCs [12, 63]. Recently, several studies have found that the sclerostin antibody has clinical implications for the treatment of osteoporosis [64, 65]. Thompson et al. [66] reported that systemic administration of sclerostin antibody in geriatric mice can enhance the proliferation of osteogenic cells in the periosteum of geriatric mice and can contribute to an increase in cortical bone thickness. Thus, the periosteum of the elderly may be an important target tissue for fracture healing and osteoporosis treatment. Although this study has shown that resveratrol can promote the osteogenesis of MSCs derived from the periosteal tissue, there are apparent limitations of its use as a treatment for osteoporosis. For example, because this study has utilized in vitro cell culture systems, a possible bone formation effect of resveratrol should be tested in in vivo models, such as geriatric animals or bone defects. Further studies aimed at an effective dose of resveratrol for in vivo animal models will also be needed for its clinical applicability and efficacy. In summary, promoting osteogenesis of PO-MSCs by a small molecule such as resveratrol can be clinically valuable for the treatment of osteoporosis and accompanying osteoporotic fracture. This study demonstrates that resveratrol, a well-known small molecule upregulating mitochondrial biogenesis can be used as a new modulator for osteogenic differentiation of PO-MSCs. Thus, this study provides a new experimental paradigm to investigate the osteogenesis of adult stem cells and the role of mitochondrial biogenesis in regenerative medicine for the bone.

## Conclusion

In conclusion, this study demonstrates that resveratrol promotes osteogenic differentiation and mitochondrial biogenesis of PO-MSCs. Therefore, enhancing osteogenesis of PO-MSCs by small molecules such as resveratrol may be clinically valuable for the treatment of osteoporosis and accompanying osteoporotic fracture.

## Data Availability

The materials described in the manuscript will be available to all scientists for non-commercial purposes.

## Abbreviations

ALP: Alkaline phosphatase
BM-MSC: Bone marrow-derived mesenchymal stem cell
DMEM: Dulbecco’s modified Eagle’s medium
OM: Osteogenesis induction medium
PO-MSCs: Periosteum-derived mesenchymal stem cells
